# Deep learning-enabled natural language processing to identify directional pharmacokinetic drug–drug interactions

**DOI:** 10.1186/s12859-023-05520-9

**Published:** 2023-11-01

**Authors:** Joel Zirkle, Xiaomei Han, Rebecca Racz, Mohammadreza Samieegohar, Anik Chaturbedi, John Mann, Shilpa Chakravartula, Zhihua Li

**Affiliations:** Division of Applied Regulatory Science, Office of Clinical Pharmacology, Office of Translational Sciences, Center for Drug Evaluation and Research, Food and Drug Administration, WO Bldg 64 Rm 2078, 10903 New Hampshire Ave, Silver Spring, MD 20993 USA

**Keywords:** Pharmacokinetic, Drug-drug interactions, Natural language processing, Directionality, Transformer language model

## Abstract

**Background:**

During drug development, it is essential to gather information about the change of clinical exposure of a drug (object) due to the pharmacokinetic (PK) drug-drug interactions (DDIs) with another drug (precipitant). While many natural language processing (NLP) methods for DDI have been published, most were designed to evaluate if (and what kind of) DDI relationships exist in the text, without identifying the direction of DDI (object vs. precipitant drug). Here we present a method for the automatic identification of the directionality of a PK DDI from literature or drug labels.

**Methods:**

We reannotated the Text Analysis Conference (TAC) DDI track 2019 corpus for identifying the direction of a PK DDI and evaluated the performance of a fine-tuned BioBERT model on this task by following the training and validation steps prespecified by TAC.

**Results:**

This initial attempt showed the model achieved an F-score of 0.82 in identifying sentences as containing PK DDI and an F-score of 0.97 in identifying object versus precipitant drugs in those sentences.

**Discussion and conclusion:**

Despite a growing list of NLP methods for DDI extraction, most of them use a common set of corpora to perform general purpose tasks (e.g., classifying a sentence into one of several fixed DDI categories). There is a lack of coordination between the drug development and biomedical informatics method development community to develop corpora and methods to perform specific tasks (e.g., extract clinical exposure changes due to PK DDI). We hope that our effort can encourage such a coordination so that more “fit for purpose” NLP methods could be developed and used to facilitate the drug development process.

## Background and significance

Over the past decade, there has been a surge of interest in developing natural language processing (NLP) methods to automatically extract and process information from biomedical literature (including regulatory drug labels). One such NLP application under active research is the automatic identification of drug-drug interactions (DDIs) [1]. This is driven by the high prevalence of potential DDIs that may lead to significant adverse events in clinical settings, and the rapid expansion of biomedical documents containing established DDI information in natural language format [2]. Recent advances in machine learning techniques, especially deep learning/neural networks, have made it possible to extract DDIs from biomedical documents automatically [2].

**Fig. 1:**
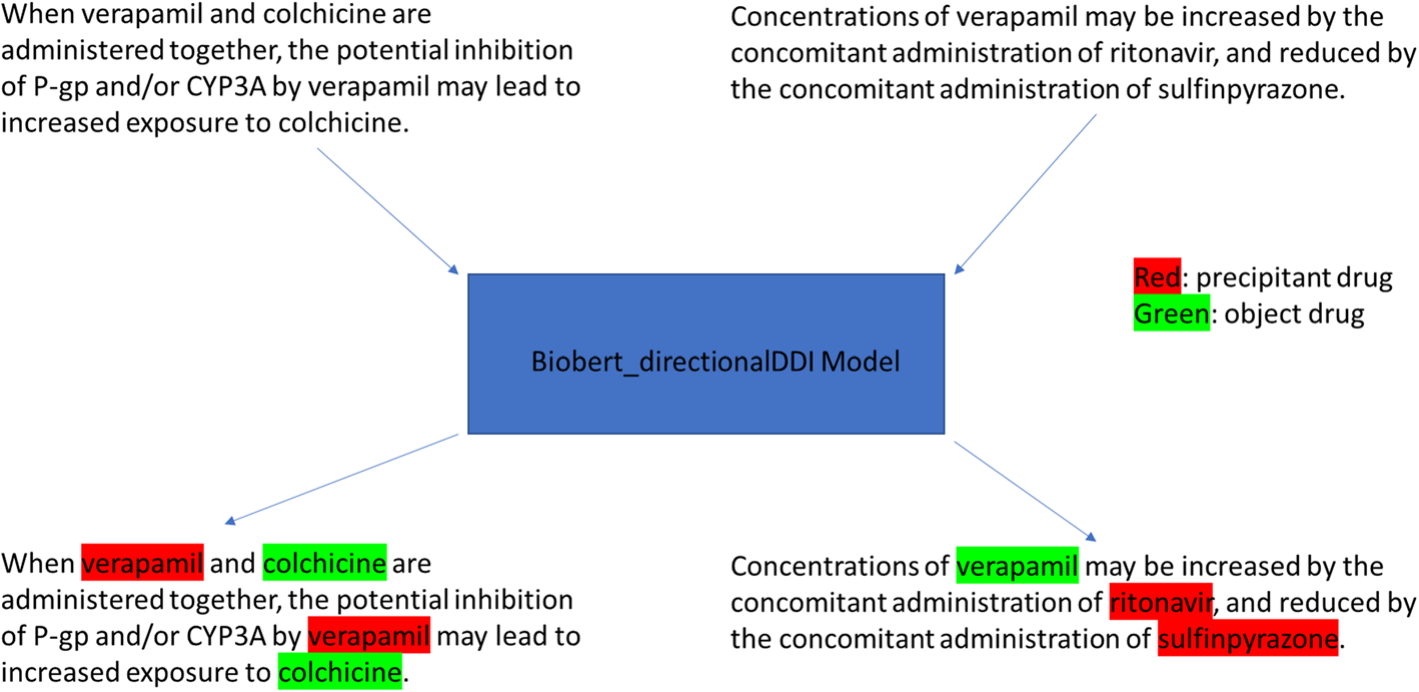
An example pair of sentences about pharmacokinetic (PK) drug-drug interaction (DDI) involving verapamil. For the left sentence, verapamil is the precipitant. For the right sentence, verapamil is the object. Our method (the BioBERT_directionalDDI model) can automatically distinguish the two sentences and label the precipitant vs object drugs

One clear example demonstrating the need for automatic methods for NLP of DDI information is the identification of the change in clinical exposures of an object drug due to other precipitant drugs (Fig. 1). This kind of pharmacokinetic (PK) DDI information is not only important in a clinical setting when prescribing medications [3], but also critical during drug development: for example, in evaluating a drug’s potential to cause QT prolongation or proarrhythmic adverse events, clinical and nonclinical studies are required by international regulatory guidelines [4] to cover the so-called high clinical exposure scenario (defined as the expected exposure when the drug is used in the presence of intrinsic or extrinsic factors, such as impaired renal function, PK DDI etc.). Given a specific drug of interest (the object drug), gathering information from existing biomedical literature and regulatory labels about all other drugs (precipitant drugs) that could change the object drug’s clinical exposure through DDI is an important step towards establishing its high clinical exposure.

There have been several initiatives that aimed at encouraging and evaluating NLP techniques to extract DDIs from biochemical literature and regulatory drug labels, for example the DDIExtraction Shared Tasks in 2011 [5] and 2013 [6], and the Text Analysis Conference (TAC) DDI tracks 2018 [7] and 2019 [8]. Various NLP methods, including traditional machine learning methods based on syntactic and lexical features, and deep learning methods based on neural networks, have been evaluated under these initiatives with varying degrees of success. However, it is difficult to apply these existing methods to the problem of automatic extraction of clinical exposure changes for object drugs due to DDI with precipitant drugs. For example, given the task of “identify all DDIs where clinical exposure of verapamil is changed by another drug from natural language text”, most published methods can only finish the first step of sentence classification: screen all sentences in literature or product labels and identify those that describe DDI relations involving verapamil. Because verapamil is both an inhibitor of cytochrome P450 enzymes and P-glycoprotein [9], and a substrate of CYP3A4 [10], there will be a large pool of sentences identified from the first step where verapamil can be either the object or precipitant drug. Consequently, in the second step most of these sentences need to be filtered out, leaving only a small subset of DDI sentences with the “correct” direction: those that describe verapamil as an object drug whose clinical exposure can be altered by other (precipitant) drugs (Fig. 1). This second step belongs to the typical NLP task of Named Entity Recognition (NER).

To the best of our knowledge the only time the task of identifying the directionality of a PK DDI was addressed was in tasks 3 and 4 of the TAC 2019 DDI track. Of the four teams that submitted methods, only one team attempted task 4 [8]. However, it does not appear that these methods were made publicly available. As such, currently there does not appear to be any published NLP method to automatically identify the direction of a PK DDI from natural language text.

### Objective

Here we report the development of a complete solution to finish both steps through NLP. Our method is based on the state-of-the-art pre-trained neural network language model BERT (Bidirectional Encoder Representations from Transformers) [11]. We manually annotated a corpus to label object versus precipitant drugs, and then fine-tuned a previously published BERT model that was pre-trained on biomedical literature (BioBERT, see [12]). We have named the resulting model BioBERT_directionalDDI, and it is designed to finish the two steps sequentially: first identify a sentence that involves PK DDI, and then label the object drug versus precipitant drug in that sentence. Of note the first step of our procedure classifies sentences into one of the relation categories without identifying which entities in the sentence have such a relation. In comparison, relation extraction (RE) tasks in the literature usually identifies relation categories associated with entities in sentences, with the entities pre-identified and anonymized [2, 12–14]. This makes our sentence classification task (1st step of our procedure) similar to the RE tasks in the sense that a relation category is identified, but identifying which entities are involved in this relation is not part of the task. The 2nd step of our procedure will complete this NER task.

Our model has enabled the efficient evaluation of high clinical exposures for some reference drugs during the development of international guidelines for cardiac safety [4], and is expected to play an important role in drug development activities where gathering information about specific drugs’ clinical exposure changes due to DDI with other precipitant drugs is necessary.

## Methods

### Datasets

The TAC 2019 DDI track [8] provided 4 training datasets: (1) 22 FDA labels fully annotated and used for TAC 2018 training, (2) Additional 180 FDA labels reannotated according to the TAC 2018 guideline, (3) 57 FDA labels used for TAC 2018 testing, (4) Additional 66 FDA labels with only the Drug Interactions and Clinical Pharmacology sections annotated. The labels were provided as Structured Product Labeling (SPL) documents in XML format, where sections and sentences were annotated according to prespecified guidelines (https://bionlp.nlm.nih.gov/tac2019druginteractions/). The combined set of training data has 21,593 sentences, each annotated as one of the 4 categories: no DDI, PK DDI, PD (pharmacodynamic) DDI, or unspecified DDI. For the purpose of our model, the no DDI, unspecified DDI, and PD DDI categories were combined into a single category of “other or no DDI”. These sentences labeled as two categories (“PK DDI” vs. “other or no DDI”) were used as training data for the first step (PK DDI sentence classification). On top of sentence-level annotations, each of these sentences also has entity-level annotation. The original XML files annotated entities of Precipitant, Trigger, and SpecificInteractions. For our model, we need Precipitant and Object entities annotated. Of note the original XML files used a definition of Precipitant that is different from ordinary DDI definitions: any drug X involved in a DDI with the labeled drug (the drug the XML file is a SPL document for) was annotated as Precipitant, even if the labeled drug actually affects drug X’s PK or PD (i.e. drug X is actually the Object drug). The third task of TAC 2019 DDI was the normalization of sentences involving PK DDI to National Cancer Institute (NCI) Thesaurus codes. Hence each PK sentence contains an NCI code label from which the correct object and precipitant drugs can be identified. We have reannotated the entities in each sentence so that the correct definition of object and precipitant is used, without having to refer to NCI codes. The resulting dataset is marked following Inside-Outside-Beginning2 (IOB2) format to indicate the boundaries of object and precipitant drugs in each sentence and used as the training data for the second step (identifying precipitant and object drugs).

**Fig. 2:**
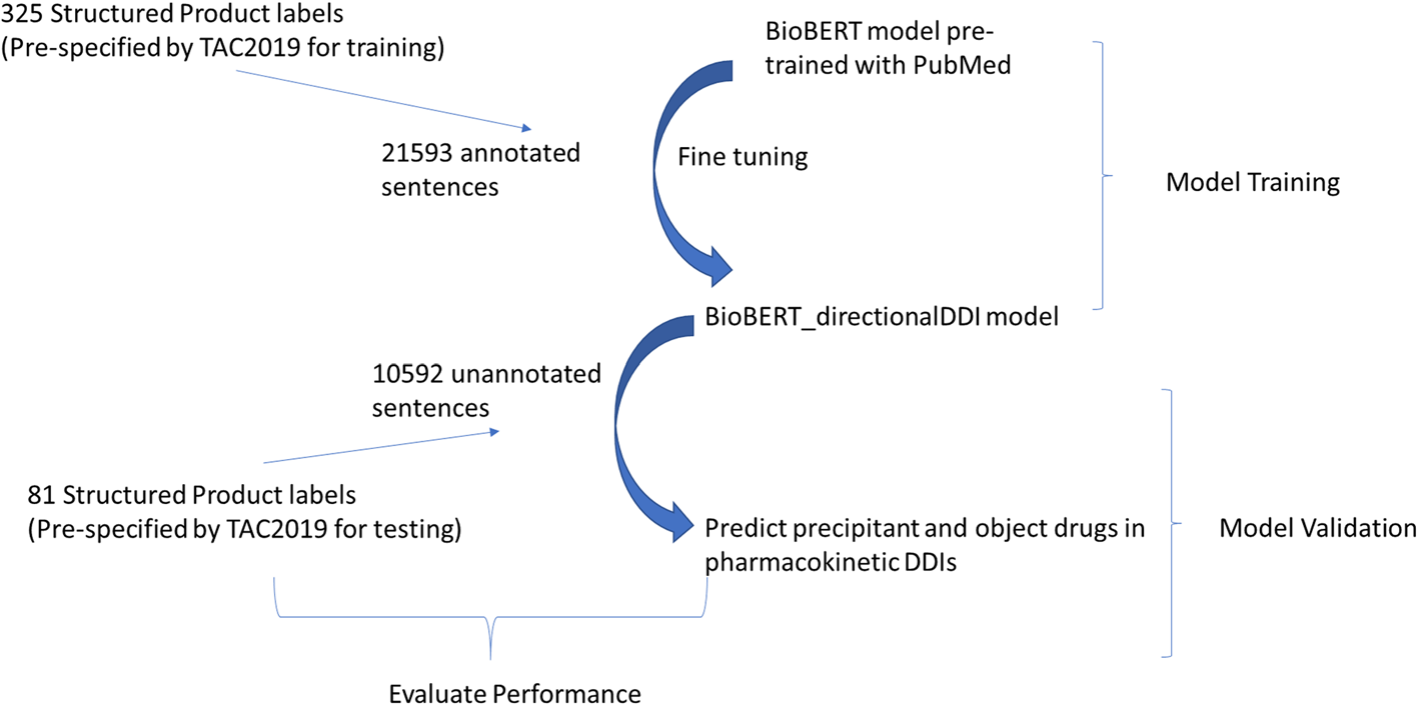
Training and validation procedure. 325 and 81 FDA labels prespecified by TAC DDI 2019 [8] were used for model training and validation, respectively. These labels were provided as Structured Product Labeling (SPL) documents as XML files. Sentences were extracted from the XML files and re-annotated to fit the purpose of the two steps of our model (DDI relation extraction to identify PK DDI sentences, and precipitant/object entity recognition in those sentences). This training/validation procedure was applied twice, each for one step of the model

Separately, the TAC 2019 DDI track provided 1 dataset containing 81 FDA labels as testing/validation data. Following the steps above, 10,592 sentences were extracted and reannotated from the XML files and used as independent validation to check the performance of our model for both steps. A diagram of the training and validation procedure can be found in Fig. 2.

### Transformer-based large language model

BERT is a recently proposed pre-training language representation model with a transformer-based large language model architecture that has demonstrated state-of-the-art results on a series of NLP tasks [11]. Building on top of BERT, Lee et al. developed BioBERT, a BERT model retrained on large scale biomedical corpora [12]. We used BioBERT-Large v1.1, which was developed by pre-training BERT-large architecture (24 layers of neural networks, 340 million parameters) on PubMed abstracts (4.5 billion words, letter case preserved) for 1 million steps, with a custom 30,000 word vocabulary (https://github.com/dmis-lab/biobert). The pre-trained BioBERT weights in the format of TensorFlow version 1 (https://www.tensorflow.org/) were downloaded from the above GitHub repository. To convert TensorFlow version 1 weights to version 2, a tf1–tf2 convert script from https://github.com/tensorflow/models/tree/r2.1.0/official/nlp/bert was used. These converted weights were loaded into an in-house developed TensorFlow version 2 implementation of BERT, modified from https://github.com/kamalkraj/BERT-NER-TF. The preloaded model was then trained (fine-tuned) to finish the two steps of the task: relation extraction (RE) to identify PK DDI sentences and named entity recognition (NER) to identify precipitant and object drugs in each PK DDI sentence. This trained neural network, referred to as BioBERT_directionalDDI, and its performance was subsequently evaluated using validation data.

For the first step of the task, the BioBERT_directionalDDI model was fine-tuned on the training data containing sentences in two categories (PK DDI and other or no DDI; see Datasets section above) with epoch size 2 and max_seq_len 128. For the second step of the task, the model was fined-tuned on the training data where precipitant and object drugs are labeled as named entities (see Datasets section above) with epoch size 50 and max_seq_len 128. Generally, we used the same hyperparameters as given in the BioBERT GitHub repository. The only difference is that we found that 2 epochs for the first step was sufficient (instead of 3 epochs as originally used in the BioBERT repository). For both steps, multiple independent models were run from random seeds to ensure that the model performance was not an outlier. It was found that the model performance was stable and so the results from a single model are presented.

In addition to using traditional classification performance metrics like precision, recall, and F score to evaluate model performance, we also performed a systematic error analysis by manually going through each wrongly predicted sentence (for step 1) or precipitant/object entity (for step 2) as an attempt to understand why the model makes a mistake. Although there were no pre-defined error categories, we noticed that most mistakes can be categorized to one of a few reasons. And we have listed a few example mistakes for each error category to facilitate discussion (see Discussion section).

### Using the model to scan all FDA prescription drug labels

The set of all human prescription drug labels was downloaded from the NIH website (https://dailymed.nlm.nih.gov/dailymed/spl-resources-all-drug-labels.cfm) on 3/15/2023 in XML format and then processed to extract all sentences. Note that the majority of text is drawn from the lists and paragraph nodes in the XMLs, however text occurring in tables is not included. Any text that is contained inside of an image was likewise not extracted. Finally, some post-process cleaning of the extracted sentences was performed, for example removal of special characters like bullet points, concatenating items in lists into a single sentence, and removing hyperlinked references.

After processing, we extracted all sentences containing one of the 28 drug names of interest (see Results) and created a data set of sentences for each drug. Then we ran our model on each drug’s data set and found all sentences that contain PK DDI information as well as all sentences where that drug appears as the object in the PK DDI. Lastly some custom scripts were used to delete redundant sentences and identify those sentences where some quantitative information were mentioned as the consequence of the PK DDI (e.g., the Cmax of a drug of interest was increased by X% when co-administered with drug Y).

## Results

### Model development using pre-specified training and validation datasets

We followed the pre-specified data split for training and validation from TAC 2019 DDI track (see Methods). Three hundred and twenty-five annotated FDA drug labels were used for model training, and 81 labels were set aside for model validation. In total there are 21,593 and 10,592 sentences for training and validation, respectively (Fig. 2). As the BioBERT_directionalDDI model contains two sequential sub-models for the two steps (relation extraction RE followed by named entity recognition NER), the performance evaluation (using the 10,592 sentences in the validation dataset) also has two sequential steps: first evaluate the accuracy of classifying all sentences into PK DDI and other or no DDI categories, then evaluate the accuracy of classifying object and precipitant drug entities in the PK DDI sentences. We report the precision, recall and F-score for both steps.

### Model performance of the first step (identifying PK-DDI sentences)

For the sentence classification task, our BioBERT_directionalDDI model resulted in a precision of 82.7%, a recall of 80.6% and an F-score of 81.6% (Table 1). This suggests that, for all sentences that actually carry PK DDI information, about 81% will be correctly classified by the model while the remaining 19% will be mistakenly classified as other or no DDI (meaning either no DDI information or DDI of other types such as pharmacodynamics).

**Table 1:** Performance of the 1st step (sentence classification to identify PK DDI)

Recall	80.6%
Precision	82.7%
F-score	81.6%

### Model performance of the second step (identifying object vs precipitant drugs in PK-DDI sentences)

For the second step (identifying object vs precipitant drugs in PK DDI sentences) our BioBERT_directionalDDI model resulted in a precision of 100% for both object and precipitant entities (there were no false positives). The recall for object entities was 93.7% and for precipitant entities it was 94.6%. The F-score for object entities was 96.7% and for precipitants entities it was 97.2% (Table 2). Therefore about 94% of all entities (object and precipitant combined) are correctly identified by the model. Such high precision and recall suggest that, given a PK DDI sentence, it is very likely that this model will correctly identify the object and precipitant drugs.

**Table 2:** Performance of the 2nd step (named entity recognition to identify precipitant and object drugs)

	Object (%)	Precipitant (%)
Recall	93.7	94.6
Precision	100	100
F-score	96.8	97.2

## Model application to identify clinical exposure changes due to DDI

Next, we applied the model to a specific use case: identify DDI-mediated clinical exposure changes of some reference drugs that were proposed to support the development of new cardiac safety regulatory guidelines [15]. The results for each of the 28 reference drugs after scanning all FDA labels for prescription drugs are shown in Table 3. The number of sentences mentioning the reference drugs ranges from around 150 (Bepridil) to over 30,000 (Quinidine). After applying the two-step approach with the model, most of the reference drugs have anywhere between a few to over a hundred unique sentences identified where the drug appears as the object in a PK DDI. These sentences form the knowledge base that was used to provide evidence and facilitate discussion for the high clinical exposure scenario of the drug.

**Table 3:** Results from BioBERT_directionalDDI applied to all human prescription drug labels from the NIH

Drug	Total sentences	PK DDI sentences	Object sentences (Unique sentences)
Vandetanib Example Sentence	923	132	16 (4)
	The coadministration of rifampicin with CAPRELSA decreased the geometric mean AUC0-504h of vandetanib by 40% (90% confidence interval (CI) 56%, 63%) compared to vandetanib alone		
Sotalol Example Sentence	16,658	121	42 (9)
	Telithromycin has been shown to decrease the Cmax and AUC of sotalol by 34% and 20%, respectively, due to decreased absorption		
Quinidine Example Sentence	31,266	7682	1583 (118)
	Diltiazem significantly increases the AUC of quinidine by 51%, T1/2 by 36%, and decreases its CL by 33%		
Ibutilide	688	0	0 (0)
Dofetilide Example Sentence	8346	645	601 (60)
	Cimetidine at 400 mg BID (the usual prescription dose) co-administered with TIKOSYN (500 mcg BID) for 7 days has been shown to increase dofetilide plasma levels by 58%		
Disopyramide Example Sentence	6087	101	49 (12)
	In vitro metabolic studies indicated that disopyramide is metabolized by cytochrome P450 3A4 and that inhibitors of this enzyme may result in elevation of plasma levels of disopyramide		
Bepridil	149	0	0 (0)
Azimilide	0	0	0 (0)
Terfenadine Example Sentence	6135	1098	629 (29)
	In a drug interaction study in 16 healthy volunteers, co-administration of multiple doses of terfenadine (60 mg every 12 h) and ZYFLO (600 mg every 6 h) for 7 days resulted in a decrease in clearance of terfenadine by 22% leading to a statistically significant increase in mean AUC and Cmax of terfenadine of approximately 35%		
Risperidone Example Sentence	68,070	3783	2250 (188)
	Venlafaxine administered under steady-state conditions at 150 mg/day slightly inhibited the CYP2D6-mediated metabolism of risperidone (administered as a single 1 mg oral dose) to its active metabolite, 9-hydroxyrisperidone, resulting in an approximate 32% increase in risperidone AUC		
Pimozide Example Sentence	13,493	2647	1818 (136)
	In a controlled study of a single dose (2 mg) of pimozide, 200 mg sertraline (q.d.) coadministration to steady state was associated with a mean increase in pimozide AUC and Cmax of about 40%, but was not associated with any changes in EKG		
Ondansetron Example Sentence	77,681	2511	1234 (24)
	In a pharmacokinetic study of 10 healthy subjects receiving a single-dose intravenous dose of ondansetron 8 mg after 600 mg rifampin once daily for five days, the AUC and the t1/2 of ondansetron were reduced by 48% and 46%, respectively		
Droperidol	1939	16	0 (0)
Domperidone	0	0	0 (0)
Clozapine Example Sentence	16,343	2774	1773 (118)
	Following concomitant administration of 250 mg CIPRO with 304 mg clozapine for 7 days, serum concentrations of clozapine and N-desmethylclozapine were increased by 29% and 31%, respectively		
Clarithromycin Example Sentence	90,366	10,775	2464 (176)
	Concomitant administration of fluconazole 200 mg daily and clarithromycin 500 mg twice daily to 21 healthy volunteers led to increases in the mean steady-state clarithromycin Cmin and AUC of 33% and 18%, respectively		
Cisapride Example Sentence	6433	1167	1001 (51)
	Following a single dose of fluconazole, there was a 101% increase in the cisapride AUC and a 91% increase in the cisapride Cmax		
Chlorpromazine	12,207	1103	101 (0)
Astemizole Example Sentence	2431	250	244 (15)
	Concomitant administration of fluconazole with astemizole may decrease the clearance of astemizole		
Verapamil Example Sentence	43,977	7691	1504 (73)
	The AUC and Cmax for both verapamil and norverapamil are increased when 240 mg of controlled release verapamil is administered concomitantly with 4 mg trandolapril		
Tamoxifen Example Sentence	27,560	961	401 (41)
	Data from a clinical trial in patients with breast cancer indicated that tamoxifen Cmax and AUC increased approximately twofold following coadministration of 600 mg ribociclib		
Ranolazine Example Sentence	11,979	1932	620 (100)
	Plasma levels of ranolazine with RANEXA 1000 mg twice daily are increased by 50 to 130% by diltiazem 180 to 360 mg, respectively		
Nitrendipine	190	0	0 (0)
Nifedipine Example Sentence	40,859	6850	3714 (177)
	The mean values of Cmax and AUC of nifedipine are increased by 64% and 79%, respectively, by co-administration of propranolol		
Mexiletine Example Sentence	5359	725	252 (20)
	In a formal, single-dose interaction study (n = 6 males) the clearance of mexiletine was decreased by 38% following the coadministration of fluvoxamine, an inhibitor of CYP1A2		
Metoprolol Example Sentence	67,595	3986	2790 (152)
	Administration of 20 mg/day Lexapro for 21 days in healthy volunteers resulted in a 50% increase in Cmax and 82% increase in AUC of the beta-adrenergic blocker metoprolol (given in a single dose of 100 mg)		
Loratadine Example Sentence	5875	436	194 (14)
	Loratadine, a non-sedating antihistaminic, is metabolized primarily by CYP3A and its metabolism can be inhibited by amiodarone		
Diltiazem Example Sentence	45,605	10,797	1830 (96)
	A study in six healthy volunteers has shown a significant increase in peak diltiazem plasma levels (58%) and area-under-the-curve (53%) after a 1-week course of cimetidine at 1200 mg per day and a single dose of diltiazem 60 mg		

The first column indicates the drug of interest. The second column (total sentences) shows the total number of sentences that the drug of interest appears in. The third column (PK DDI sentences) shows the number of sentences where the drug appears that also contain some PK DDI information. The fourth column (object sentences) shows the total number of sentences where the drug of interest appears as the object in the PK DDI. There is a, potentially large, number of repeated sentences for each drug across all the drug labels so the number in parentheses in this column indicates the number of unique sentences. Of note two of the reference drugs, azimilide and domperidone, did not have any sentences mentioning their names probably because they were not approved in the USA. And for some other drugs, such as bepridil, that were mentioned in sentences in their own labels or other drugs’ labels, however none of these sentences contain PK DDI. Where possible, the second row for a drug shows an example sentence identified by the model. Most of these example sentences have quantitative information that can help to facilitate the determination of the high clinical exposure of the reference drug

## Discussion and conclusion

### Background of project initiation

In this paper we reported the development of a transformer-based large language model to automatically identify precipitant and object drugs involved in a PK DDI relation. This project was started during the development of international cardiac safety regulatory guidelines where the change of clinical exposure of a drug (object) due to DDI with another drug (precipitant) needs to be considered to assess the “high clinical exposure” of the object drug. We were surprised by the lack of automatic solutions (either commercial or open source) to this important task, and decided to develop the current model (BioBERT_directionalDDI) by manually annotating a corpus and then fine tuning the state-of-the-art language model BERT [11].

### A comprehensive and properly annotated corpus to identify precipitant and object drugs

To identify the clinical exposure change due to PK DDI from a sentence there are naturally two steps: first to identify those sentences that carry DDI information in the PK category, then to identify the precipitant and object drugs in those sentences. Almost all published NLP methods were designed to finish the first step only. The lack of existing methods to tackle the second step of identifying the directionality of the PK DDIs could be due to the lack of a large and properly annotated corpus for this task. It’s worthwhile to acknowledge that creating such a corpus is not a simple task as it may require dealing with sentences where the PK DDI is bi-directional or is ambiguously worded and the annotator will have to deal with these cases in a consistent manner. To the best of our knowledge there are only two corpora with the proper annotations of object and precipitant in the context of PK DDIs: the PK DDI corpus from Boyce et al. [16] and TAC 2019 DDI corpus (after translating the associated NCI codes). However, the Boyce corpus was based on only 64 product labels, and only 1 to 2 selected sections from each label were extracted and annotated. In contrast, the TAC 2019 DDI corpus we re-annotated was from 406 product labels (training and validation combined), and for most of these labels the entire documents were annotated. Probably because of the small amount of data available for training, even though their corpus contains the annotations of object and precipitant for PK DDIs, Boyce’s methods were only built to detect PK DDIs and their “modality” but not identify the objects or precipitants [16]. Another well-known DDI corpus from Herrero-Zazo et al. [1] identifies DDIs of the PK category (through the type “mechanism”) and annotates the entities involved in this PK DDI. However, the entities are labeled in the sequence they appear in the sentence, not for their functionality in the DDI (i.e. not as precipitant or object). We decided to re-annotate the TAC 2019 DDI corpus with the entities of precipitant and object readily identified (without recourse to NCI codes) for ease of use in our method. This corpus was then used in our training and validation process.

### Fine-tuning existing BERT-based language models achieved reasonable performance

In the beginning of our project we searched for available methods that can identify PK DDI sentences and the associated precipitants/objects. The only published method that can potentially finish both steps is from the Human Language Technology Research Institute (HLTRI) at the University of Texas at Dallas (UTD) as a participating team for TAC 2019 [17]. However, their method predicts NCI codes, which will need to be further translated to precipitant/object relationships. And to the best of our knowledge, the method is not open sourced, making it hard to reapply their method to our corpus to evaluate or compare performance. In the absence of state-of-the-art or reference solutions, we fine-tuned the pretrained model BioBERT-Large v1.1 [12] on our annotated training datasets directly, without trying to modify the model structure to further improve the performance. We used traditional classification performance metrics like precision and recall, as well as F score, to assess the accuracy of the model. Based on the validation datasets prespecified by the TAC 2019 DDI track (and newly annotated by us, see above and Methods), our model has an F-score of 0.82 in identifying PK DI sentences (first step) and an F-score of 0.97 in identifying object vs precipitant drugs (second step). Of note the last layer of our neural network is a softmax layer that will produce the probability of the input sample being in each of the categories. For example, after the 1st step, each sentence will be assigned a probability X (0 < X < 1) to be in “PK-DDI” category and 1-X to be in “other or no DDI” category. Since X is a continuous variable, in theory one could use Receiver Operating Characteristic (ROC) curves to illustrate the performance over the whole range of possible classification thresholds (which is the range of X) and pick a threshold for maximum performance. We used a simpler “maximum argument” approach that essentially fix the classification thresholds of X to be 0.5, as this approach is widely used in the machine learning literature adopting neural networks for classification [2, 11, 12].

### Error analysis

For the first step, a detailed investigation into the false negatives revealed several reasons for missing some of the PK DDI sentences.

Sometimes the sentence itself does not contain enough information to be classified as PK DDI (Table 4A). For example, the sentence “Griseofulvin decreases the activity of warfarin-type anticoagulants so that patients receiving these drugs concomitantly may require dosage adjustment of the anticoagulant during and after griseofulvin therapy” was manually annotated as (and hence has a true label of) PK DDI in the validation dataset. Although it is generally accepted that griseofulvin decreases warfarin activities through PK mechanisms such as inducing metabolizing enzymes and interfering with absorption [18], such information is not contained in the sentence above that was presented to the model. This explains why the model misclassified it as other or no DDI.

**Table 4:** Representative false negative (A) and false positive (B) sentences for the first step (relation extraction to identify PK DDI sentences)

Sentence in Validation Dataset	True Label	Predicted Label	Reason for Wrong Prediction
*A*			
Griseofulvin decreases the activity of warfarin-type anticoagulants so that patients receiving these drugs concomitantly may require dosage adjustment of the anticoagulant during and after griseofulvin therapy	1	0	Sentence does not contain information to be classified as PK DDI
Barbiturates usually depress griseofulvin activity and concomitant administration may require a dosage adjustment of the antifungal agent	1	0	Sentence does not contain information to be classified as PK DDI
Elimination can be accelerated by the following procedures: 1)Administer cholestyramine 8 g orally 3 times daily for 11 days	1	0	The name of the drug for the drug label is omitted from the sentence in some old drug labels
There may be competition for elimination with other compounds that are also renally eliminated	1	0	The name of the drug for the drug label is omitted from the sentence
*B*			
The glucose lowering effect of ADMELOG may be decreased when co-administered with corticosteroids, isoniazid, niacin, estrogens, oral contraceptives, phenothiazines, danazol, diuretics, sympathomimetic agents (e.g., epinephrine, albuterol, terbutaline), somatropin, atypical antipsychotics, glucagon, protease inhibitors, and thyroid hormones	0	1	Sentence contains information to be classified as DDI, but not enough information to distinguish PK vs non-PK mechanisms
Compared to a patient with a body weight of 65 kg, the rivastigmine steady-state concentrations in a patient with a body weight of 35 kg would be approximately doubled, while for a patient with a body weight of 100 kg the concentrations would be approximately halved	0	1	Sentence contains information about an interaction between drugs and non-drug elements (body weight)
Smoking Following oral rivastigmine administration (up to 12 mg/day) with nicotine use, population pharmacokinetic analysis showed increased oral clearance of rivastigmine by 23% (n = 75 smokers and 549 nonsmokers)	0	1	Sentence contains information about an interaction between drugs and non-drug elements (smoking)
Intervention: Dose reductions and increased frequency of glucose monitoring may be required when BASAGLAR is co-administered with these drugs	0	1	Sentence contains information to be classified as DDI, but not enough information to distinguish PK vs non-PK mechanisms

Another reason is unique to some documents in the validation dataset: each document is the label of a specific FDA-approved drug (which is referred to as “label drug” hereafter), and in some sections of some old labels the name of the label drug is omitted from a sentence (Table 4A). For example, the sentence “Elimination can be accelerated by the following procedures: 1) Administer cholestyramine 8 g orally 3 times daily for 11 days” does convey the DDI information between cholestyramine and some other drug. The other drug is leflunomide (Arava), which is the label drug and hence is omitted from the sentence. Consequently, the model did not classify it as a PK DDI sentence. This kind of sentence is a unique feature of old drug labels and is unlikely to be encountered when examining more recent drug labels or literature in scientific journals.

We also performed a similar error analysis for false positives (Table 4B). Some sentences were mistakenly classified as PK DDI because they contain information about interaction between a drug and a non-drug factor (e.g. body weight or smoking). This can be seen from the sentence “Smoking: Following oral rivastigmine administration (up to 12 mg/day) with nicotine use, population pharmacokinetic analysis showed increased oral clearance of rivastigmine by 23% (n = 75 smokers and 549 nonsmokers)”. In addition, there are also some sentences that do not carry enough information to be classified as PK DDI or other or no DDI by themselves, such as “Intervention: Dose reductions and increased frequency of glucose monitoring may be required when BASAGLAR is co-administered with these drugs”. Overall, we calculated the specificity of the model on the sentence classification step and found that it was extremely high; about 0.99, this indicates that the fraction of other or no DDI sentences that are wrongly classified as PK DDI is small.

Error analysis of the second step (Table 5) suggests that some object/precipitant classifications were wrong because the corresponding drug names appear in the sentence in a complex way. For example, in the sentence: “In patients taking ARAVA, exposure of drugs metabolized by CYP1A2 (e.g., alosetron, duloxetine, theophylline, tizanidine) may be reduced”, the model correctly identified that ARAVA is the precipitant drug while alosetron, duloxetine, theophylline, and tizanidine are the object drugs. However, the original sentence also labeled “drugs metabolized by CYP1A2” as a general term to cover object drugs, which the model missed. Notice that this example shows that the model can handle situations where there are multiple entities of the same class; in this case there are multiple object drugs. There are also other object/precipitant drugs that were misclassified without obvious reason (Table 5). But overall, the high precision and recall (both > 0.9) indicate that these wrongly classified directional DDI entities are relatively rare.

**Table 5:** Representative examples where precipitant and/or object drugs were missed by the model during validation of the 2nd step (named entity recognition to identify precipitant and object drugs)

Sentence	True label	Predicted label	Reason for wrong prediction
Barbiturates usually depress griseofulvin activity and concomitant administration may require a dosage adjustment of the antifungal agent	Precipitant: Barbiturates Object: griseofulvin	Precipitant: None Object: griseofulvin	Missing precipitant: reason unknown
Potent inhibitors of CYP3A4 can increase the plasma concentrations of budesonide	Precipitant: Potent inhibitors of CYP3A4 Object: budesonide	Precipitant: inhibitors of CYP3A4 Object: budesonide	For precipitant, missing the adjective “potent”
In patients taking ARAVA, exposure of drugs metabolized by CYP1A2 (e.g., alosetron, duloxetine, theophylline, tizanidine) may be reduced	Precipitant: ARAVA Object: drugs metabolized by CYP1A2, alosetron, duloxetine, theophylline, tizanidine	Precipitant: None Object: ARAVA, alosetron, duloxetine, theophylline, tizanidine	ARAVA (a precipitant) is mislabeled as object: reason unknown Missing “drugs metabolized by CYP1A2” as object: not a specific drug name
Intervention: Mycophenolate mofetil (MMF): Co-administration of PPIs in healthy subjects and in transplant patients receiving MMF has been reported to reduce the exposure to the active metabolite, mycophenolic acid (MPA), possibly due to a decrease in MMF solubility at an increased gastric pH	Precipitant: PPIs Object: Mycophenolate mofetil, mycophenolic acid	Precipitant: PPIs Object: Mycophenolate mofetil	Missing one object: mycophenolic acid is a metabolite
Caution should be exercised when considering the coadministration of ASMANEX HFA with ketoconazole, and other known strong cytochrome P450 (CYP) isoenzyme 3A4 (CYP3A4) inhibitors (e.g., ritonavir, cobicistat-containing products, atazanavir, clarithromycin, indinavir, itraconazole, nefazodone, nelfinavir, saquinavir, telithromycin) because adverse effects related to increased systemic exposure to mometasone furoate may occur	Precipitant: ketoconazole, ritonavir, cobicistat-containing products, atazanavir, clarithromycin, indinavir, itraconazole, nefazodone, nelfinavir, saquinavir, telithromycin Object: ASMANEX HFA, mometasone furoate	Precipitant: missing ketoconazole, ritonavir, and itraconazole Object: missing ASMANEX HFA	Unknown

### Potential model application use cases

As mentioned earlier this model was developed to facilitate the gathering of high clinical exposure information for reference drugs during the discussion of cardiac safety regulatory guidelines [4]. In addition, our model could be used in specific drug development program when the drug of interest has relevant information in other drug labels or scientific literature. For example, a comprehensive scanning of all drug labels and/or literature to gather information about DDI-associated clinical exposure increase of a drug of interest could potentially be used to help the selection of a target clinical exposure for this drug in a first-in-human QT assessment to fulfill the International Council for Harmonisation (ICH) E14 Q & A 5.1 requirement [4]. And natural text mining using the model could be used for post marketing pharmacovigilance surveillance for specific drugs [19].

### Limitations

A few limitations of our method should be noted. First, there is potentially useful PK information contained in tables and figures in drug labels that our method currently cannot use. Extraction of information in these forms can be challenging, however there has been some recent work in the area [20]. Another limitation is that our method analyzes each sentence individually; whereas sometimes contextual knowledge from surrounding sentences can be useful in determining whether a sentence contains PK DDI and also its directionality. Lastly, we mention that after annotating our corpus and training our model that they are fixed in time, and may need to be updated; for instance, if changes are made to how drug interaction information is recorded.

### Potential next steps

As stated above, some classification errors are attributed to a lack of information contained in the sentence. This may require new generations of AI methods that enquire external sources during the classification steps. For example, in the case of sentences from drug labels that allude to the label drug, without explicitly naming it in the sentence, we could pull the label drug name from other parts of the drug label or from a database such as RxNorm [21]. For other classification errors where the relevant information is contained in the sentence already, they may be resolved by improving the existing BERT-based pipelines, such as supplementing the pre-training materials (which are mostly biomedical literature) with FDA drug labels, adjusting the number of layers, etc.

Even though general DDI corpora may exist, these usually can only be used to develop methods for general purpose DDI extraction (e.g., classifying a sentence into one of several DDI categories). Hence it is important that once users have defined a more specific task (e.g., identifying clinical exposure changes of object drugs due to PK DDI with precipitant drugs), they provide a specific corpus that can support the development of NLP methods to perform the task. Here we hope our model provides a temporary solution to the task of automatic identification of directional DDI from biomedical literature and drug product labels. More importantly, we hope our initial attempt can encourage the biomedical informatics method development community to engage the drug development community more to develop “fit for practical purpose” methods, and the drug development community to annotate and release high quality corpora for specific tasks they are facing in the drug development process.

## Data Availability

All scripts and datasets used can be found at https://github.com/FDA/Neural-Networks-based-Natural-Language-Processing.
